# Establishment of rat ankle post-traumatic osteoarthritis model induced by malleolus fracture

**DOI:** 10.1186/s12891-017-1821-9

**Published:** 2017-11-17

**Authors:** Dawei Liang, Jian Sun, Fangyuan Wei, Jianzhong Zhang, Pengcui Li, Yingke Xu, Xianwen Shang, Jin Deng, Ting Zhao, Lei Wei

**Affiliations:** 1 Department of Orthopaedics, The Second Hospital of Shanxi Medical University, Taiyuan, China; 2 Foot and Ankle Orthopaedic Surgery Center, Beijing Tongren Hospital, Beijing, China; 3 School of Community Health Science, Nevada Institute of Personalized Medicine, University of Nevada, Las Vegas, Nevada USA; 4 Department of Orthopaedics, Affiliated Hospital of Guizhou Medical University, Guiyang, China; 5 Department of Orthopaedics, Warren Alpert Medical School of Brown University and Rhode Island Hospital, Providence, RI USA

**Keywords:** Ankle, Post-traumatic osteoarthritis, Animal model, Fracture, Rat

## Abstract

**Background:**

Malleolar fracture, which is present in 37–53% of human ankle osteoarthritis (OA), is the most common type of fracture in the ankle joint. In spite of this, no rat animal model has been developed for this type of injury to date. Here, we established a rat ankle post-traumatic OA (PTOA) model induced by malleolar fracture; this model will be useful in ankle OA research.

**Methods:**

Two-month-old male Sprague Dawley (SD) rats were randomized into 2 groups (*n* = 19 per group): 1) malleolus articular fracture, dislocation, and immediate reduction on the right joints and 2) malleolus articular fracture on the right ankle. The contralateral ankle joints were used as controls. The fracture and healing processes were confirmed and monitored by radiography. Changes in inflammation were monitored in vivo by fluorescence molecular tomography (FMT). Cartilage damage and changes in expression of OA-related genes were analyzed by histology, immunohistochemistry, Real-time quantitative PCR (qPCR) and enzyme-linked immunosorbent assay (ELISA) at 8 weeks post-surgery.

**Results:**

X-rays showed that all fractures were healed at 8 weeks post-surgery. A reproducible, mild to moderate degree of OA cartilage damage with reduced aggrecan was detected by histology in all animals in both groups but there was no significant difference between the two groups. Decreased Col-II and increased Col-X and MMP-13 levels were detected by qPCR, immunohistochemistry, ELISA and FMT from both groups cartilage.

**Conclusions:**

Malleolus articular fracture alone induces ankle OA with lesions on the central weight bearing area of the tibiotalar joint in rats. This model will provide a reproducible and useful tool for researchers to study ankle OA.

## Background

Osteoarthritis (OA) is the most common cause of disability in the elderly [1]. Disability stems from pain and limitations in mobility secondary to the degeneration of articular cartilage, a trademark of the disease. Unfortunately, current pharmacological therapy targeting the mechanism of OA is relatively ineffective, largely because the etiology and pathogenesis of OA remain poorly understood. The complex pathobiological changes that occur in human OA may be influenced by a multitude of genetic and environmental factors. The effort to clarify the molecular events that occur in OA during the onset and the progression of OA has necessitated the use of in vivo models [2]. Researchers tend to utilize knee OA models to investigate these factors, but have neglected the establishment of other types of OA models, such as ankle OA.

A recent study indicates that the biomarker and mechanism of ankle OA may not be the same as those of knee OA [3–5]. Researchers have reported that aggrecan (Acan), bone morphogenetic protein (BMP)-2, BMP-7, and fibronectin-aggrecan complex (FAC) can be used as key markers of OA in the ankle, but not in the knee [6, 7]. In the knee and hip, primary OA accounts for 67% and 58% of all cases, respectively. Meanwhile, 78% of all cases of ankle OA are post-traumatic (PTOA) [8, 9]. In addition, while the incidence of knee OA in the adult population rises from 6% to 10% after 65 years of age, the incidence of ankle OA remains unchanged with age [10]. Malleolar fractures are the most frequent type of fracture in the ankle, presenting in 37–53% of patients with advanced or end-stage ankle OA [11, 12]. More than 50% of patients with fractures of the distal tibial articular surface develop OA [13]. After intra-articular fracture, the ankle joint sustains increased contact stress; in addition, the inflammatory response is a contributory factor to the progress of OA [14]. Chondrocyte necrosis and apoptosis are observed following trauma in human and porcine knees, and associated with cartilage damage and degeneration [15]. An advantage of PTOA models is that there is temporal control of disease induction (when compared with spontaneous animal OA and with human disease), while mimicking the molecular pathology and histopathology of human disease [2]. Despite the high incidence of ankle trauma and OA, ankle-specific OA research is sparse, with the majority of clinical and basic research pertaining to the knee and hip joints [16]. This will greatly limit the study of ankle OA. Thus, there is a need to develop novel ankle PTOA models to facilitate research of this type of OA. Clinically, some patients with malleolar fracture present only, while others present with fracture and dislocation. Therefore, we developed two rat ankle PTOA models in this study: 1) the malleolus fracture with dislocation and reduction; and 2) the malleolus fracture alone. The contralateral ankles were used as controls. To validate the success of our models, X-ray and Safranin-O were used to observe morphological changes in the subchondral bone, joint space and cartilage. FMT, ELISA and immunohistochemistry were used to detect protein levels of several OA-related biomarkers, and qPCR was used to obtain the mRNA levels of several OA related genes.

## Methods

### Experimental animals

This study was approved by the Institutional Review Board and the Institutional Animal Care and Use Committee of the Shanxi Medical University (2015LL020). Thirty-eight skeletally mature 2-month-old male Sprague Dawley (SD) rats (220 ± 20 g), which were from Shanxi Medical University Experimental Animal Center, were randomized into 2 groups (*n* = 19 per group): Group 1 (fracture + dislocation + reduction) underwent fracture of the right medial malleolus, dislocation, and immediate reduction; Group 2 (fracture alone) underwent fracture of the right medial malleolus. The contralateral ankle joints were used as controls. Animals were housed in groups of 2 rats per cage. They had free access to food and water throughout the experiment. Eight weeks after surgery, rats were euthanized with an overdose of pentobarbital sodium (150 mg/kg IV).

### Surgical fracture of medial malleolus with or without dislocation

**Fig. 1:**
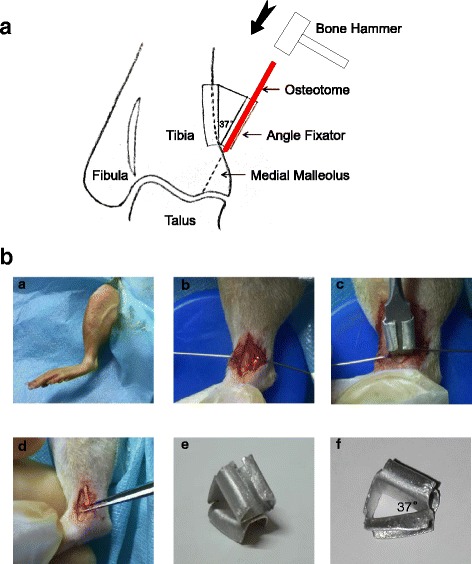
**a** Schematic illustration of surgical procedure in right ankle joint (anterior view). The dashed line in medial malleolus was the location of fracture. **b** The procedure of ankle surgery-induced OA model. **a** Position of ankle joint. **b** Exposure of medial malleolus. **c** Osteotome and angle fixator placed together on the distal tibia. **d** Micro-surgery forceps were used to make sure fracture was complete. **e**-**f** Front and lateral views of angle fixator

The site of fracture was shown in the schematic diagram of right ankle joint (Fig. 1a). The animals were anesthetized, and the ankles were prepared for aseptic surgery as before [17]. Rats were maintained on supine position with the right hip joint in 90 degree abduction, and the right knee joint bent at 90 degree. A 1-cm longitudinal incision was performed on the medial malleolus with a #11 blade. Subsequently, blunt dissection of the superficial and deep fascia and the tibialis posterior tendon was carried out in order to expose the medial malleolus. Two 1-ml syringes were used as retractors. The osteotome, combined with an angle fixator (37 degree, to create a reproducible and stable fracture located at medial 1/3 tibiotalar joint) was put in the distal tibia and peened into the medial malleolus until there was sudden stop of resistance. Micro-surgery forceps were used to clamp and wobble the fracture fragment and ensure that it was completely fractured (Fig. 1b). The dislocation group was performed by a malleolar varus (attention was paid to not injure the lateral ligament) and reduction was immediately performed. Before closing the incision, the fracture fragment was compressed to achieve anatomical restoration. The incision was closed layer by layer with 4–0 suture. These animals were allowed to move freely after surgery. The post-operative analgesia was maintained using buprenorphine hydrochloride (0.03 mg/kg SQ for three days) to relieve pain and distress. No animal was excluded in this study.

### Radiography

X-rays were taken immediately in supine position under anesthesia condition after fracture to make sure the malleolus fracture was successful. Fracture healing and OA changes were confirmed at week 8 after fracture by UltraFocus100 x-ray cabinet (Faxitron, Arizona, USA). The exposure time was 4 s and the kV settings were about 30–40 kV.

### Fluorescence molecular tomography (FMT)

FMT is a noninvasive and quantitative fluorescence-based technology with high molecular specificity and sensitivity for 3-dimensional tissue imaging of live animals. Using in vivo FMT imaging methods and probes, real-time and deep tissue imaging information can be gained about biological processes [18, 19]. In this study, FMT was used to monitor the levels of inflammation in vivo 24 h after intra-articular injection of MMPSense 680 (10 μl, 13.3 μM) (PerkinElmer, Massachusetts, USA), which detects MMPs-3, -9, and -13. The picomolar concentrations of probes in the ankle joint were determined using region of interest analysis (ROI), and restricting the area of measurement to the distal-tibia to talus in order to isolate the joint space [20]. Data are reported as means ± SD, *n* = 5/per group.

### Enzyme-linked immunosorbent assay (ELISA)

Synovial fluid (SF) lavages were immediately collected from the ankles after euthanasia. Briefly, 100 μl of isotonic saline solution was injected into the ankle joint through the front joint cavity using a 0.3-ml insulin syringe with 31G needle. The joint capsule was visibly distended after injection. Before collection the ankle was manually cycled through flexion and extension 5 times to distribute the fluid. About 70 μl of the injected fluid was recovered. These samples were centrifuged for 20 min at 1000 g and frozen at −80 °C until analysis. MMP-13 content was measured in the SF samples using ELISA Kit (Uscn Life Science, Wuhan, China) according to the instructions of the manufacturer. The samples were diluted at 1:1 in phosphate buffer saline (PBS). Colorimetric density on the developed plates was determined using a Thermo Multskan Mk3 microplate reader (Thermo, Massachusetts, USA) set to 450 nm. Data are reported as means ± SD, *n* = 8/per group.

### Histologic evaluation

Rats were humanely sacrificed 8 weeks after surgery. The ankle joints were fixed in 4% formaldehyde for 48 h. Whole joints were decalcified in 20% EDTA for 6 weeks on a shaker. Each ankle joint including the distal tibia and talus was hemisected in the mid-coronal plane, an anterior and a posterior one. The two resulting tissue pieces (anterior and posterior half) were then both embedded in a single paraffin block with the cut planes facing down. Blocks were trimmed to expose cartilage. Ten adjacent sections were collected at intervals of 0 μm, 100 μm, and 200 μm. Two serial 5-μm-thick slides from each interval were stained with Safranin-O and Fast Green. Cartilage degradation was quantified by two independent and blinded observers using a modified Osteoarthritis Research Society International (OARSI) grading system based on OARSI score [21]. The joint surface of the distal tibia and talus was respectively divided into three zones of equal width using an ocular micrometer or a ruler on a photograph. A score of 0 was given to normal cartilage; 1 for samples with 5–10% of the total projected cartilage area affected by Acan, matrix or chondrocyte loss and matrix fibrillation; 2: 11–25% affected; 3: 26–50% affected; 4: 51–75% affected; 5: greater than 75% affected. The maximum cartilage damage score is 30. Data are reported as means ± SD, *n* = 11/per group. Eleven rats were used for histologic analyses; the remaining 8 rats were used for collection of synovial fluid lavage and cartilage for ELISA and qPCR.

### Immunohistochemistry

Type II collagen, type X collagen, and MMP-13 were analyzed by immunohistochemistry using an UltraSensitive™ SP IHC Kit (Maixin Biotech, Fuzhou, China). For antigen retrieval, sections were digested with 0.05% trypsin for 20 min at 37 °C. Endogenous peroxidase activity was quenched with endogenous peroxidase block and nonspecific antibody binding was blocked by goat nonimmune serum for 10 min at room temperature. The sections were incubated with primary antibody against either rat type II collagen (Boster, Wuhan, China), type X collagen, or MMP-13 at 4 °C overnight. Thereafter, the sections were incubated with biotinylated secondary antibody and streptavidin-peroxidase conjugate each for 10 min at room temperature, then developed in 3,3′-diaminobenzidine chromogen. Photography was performed with an Olympus BX51 microscope (Olympus, Tokyo, Japan). Counting of positively stained cells was achieved using Image-Pro Plus 6.3 system at ×400 magnification. Five areas of cartilage were counted randomly and results expressed as average mean number of positive cells. Areas near chondrocyte and matrix loss were excluded. Slides were counted by two blinded and independent observers. Data are reported as means ± SD, *n* = 3/per group.

### Real-time quantitative PCR (qPCR)

Cartilage samples were scraped with #11 blade and ground with mortar and pestle under liquid nitrogen (*n* = 6). Total RNA was isolated from cartilage using a RNAiso Plus (Takara, Dalian, China). 1 μg total RNA was reverse transcribed to complementary DNA (cDNA) using a Prime Script™ RT Master Mix (Takara, Dalian, China). The resulting cDNA (40 ng/μl) was used as the template to quantify the relative level of messenger RNA (mRNA) using a SYBR Premix Ex Taq™ II (Takara, Dalian, China) with a iQ™5 Optical Module Detection System (Bio-Rad, California, USA). Primer pairs were as follows: for rat Col2a1, AAG-GGA-CAC-CGA-GGT-TTC-ACT-GG (forward) and GGG-CCT-GTT-TCT-CCT-GAG-CGT (reverse); for rat Acan, CAG-TGC-GAT-GCA-GGC-TGG-CT (forward) and CCT-CCG-GCA-CTC-GTT-GGC-TG (reverse); for rat MMP-13, GGA-CCT-TCT-GGT-CTT-CTG-GC (forward) and GGA-TGC-TTA-GGG-TTG-GGG-TC (reverse); and for 18S RNA, CGG-CTA-CCA-CAT-CCA-AGG-AA (forward) and GCT-GGA-ATT-ACC-GCG-GCT (reverse). Relative transcript levels were calculated according to the equation x = 2^-ΔΔCt^, where ΔΔCt = ΔCtE -ΔCtC (ΔCtE = CtE - Ct18S, ΔCtC = CtC - Ct18S) [17]. Data is reported as means ± SD.

### Statistical analysis

Statistical differences were assessed with two-way ANOVA with repeated measures. Follow-up pairwise comparisons were carried out using the Bonferroni post-test. Results were expressed as the mean ± SD, and *P* values smaller than 0.05 were considered statistically significant. Statistical analysis was performed with GraphPad Prism 5 software.

## Results

### Radiography

**Fig. 2:**
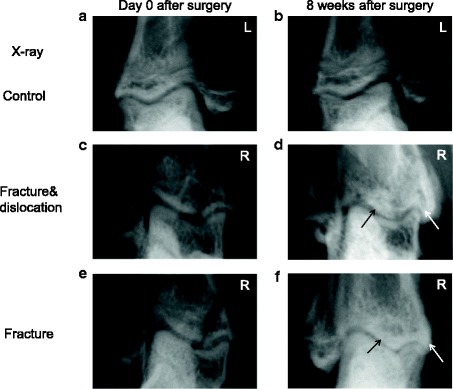
Radiography demonstrated the OA changes in the ankle joint 8 weeks after surgery (**b, d,** and **f**) when compared with immediately after surgery (**a, c**, and **e**). **d** and **f** suggested that the joint space became narrow. Black arrow shows subchondral sclerosis and white arrow shows osteophytes

The medial malleolus fracture was confirmed in all rats (Fig. 2c and e). X-ray showed that all fractures were healed completely 8 weeks after surgery (Fig. 2d and f). The joint space in the fracture groups was narrow compared to that on day 0 after surgery and the control groups, and subchondral sclerosis and osteophytes appeared at week 8 after surgery (Fig. 2).

### Fluorescence molecular tomography (FMT)

**Fig. 3:**
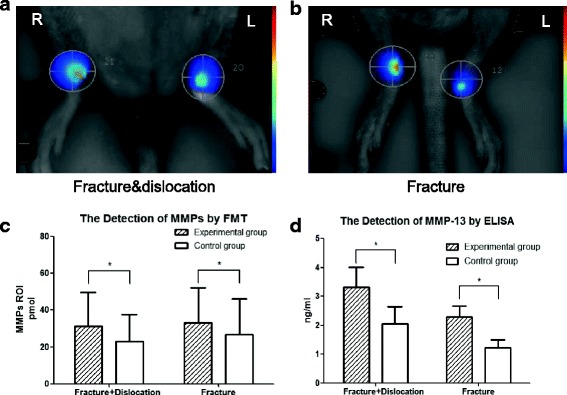
MMP-13 was detected by FMT and ELISA. FMT indicated that the positive MMPs signals were enhanced in the fractured ankles 8 weeks after surgery. **a** and **b** showed images of FMT signals. **c** showed quantitative FMT data. Values are the mean ± SD, * = *P* < 0.05 versus controls. **d** The concentration of MMP-13 detected by ELISA indicated that MMP-13 levels in the surgical sides were higher than those in the control. Values are the mean ± SD, * = *P* < 0.05 versus controls

FMT data indicated that MMPs were higher in the operated ankles than in the contralateral sides 8 weeks after fracture. MMPs in the dislocation group vs control were 31.36 ± 18.19 pmol vs 23.05 ± 14.49 pmol (mean ± SD, *n* = 5), *t* = 4.382, *P* < 0.05. Similarly, MMPs in the fracture group vs control were 33.02 ± 19.19 pmol vs 26.70 ± 19.35 pmol, *t* = 3.328, *P* < 0.05 (Fig. 3a and b). Detailed data are shown in Fig. 3c. There was no significant difference in MMPs between the dislocation and fracture-alone animals (*F* = 0.056, *P >* 0.05).

### Enzyme-linked immunosorbent assay (ELISA)

The MMP-13 concentration in SF lavages as detected by ELISA was 3.33 ± 1.93 ng/ml in the dislocation group (mean ± SD, *n* = 8) and in the control it was 2.05 ± 1.69 ng/ml (*t* = 3.348, *P* < 0.05). The level of MMP-13 in the fracture group was 2.30 ± 1.07 ng/ml and in the control it was 1.23 ± 0.75 ng/ml (*t* = 2.792, *P* < 0.05) (Fig. 3d). Similarly, no differences were detected by ELISA between the dislocation and fracture-alone animals (*F* = 1.928, *P >* 0.05).

### Histologic evaluation

**Fig. 4:**
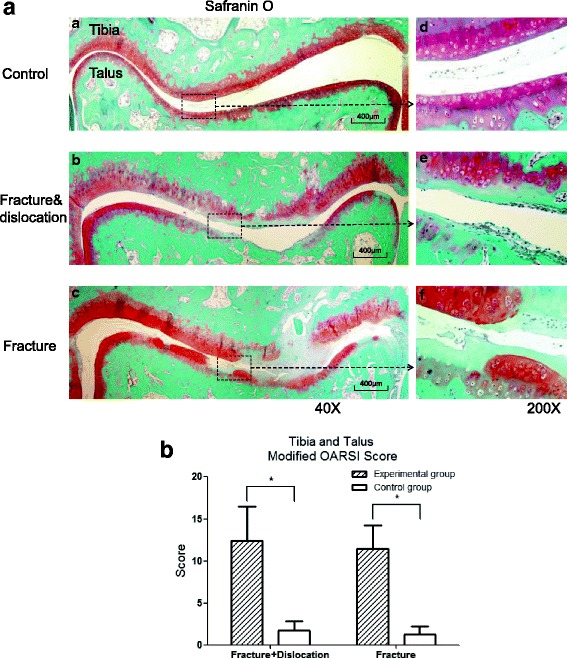
Safranin-O staining and quantification of the histological results. **a** Unlike the control groups (**a** and **d**), OA changes were observed in fracture groups, including cartilage fibrillation and cranny, rough articular surface, the loss of Acan, matrix and chondrocytes (**b-c** and **e-f**). Particularly, OA lesions were more severe in the central cartilage when compared with the peripheral cartilage. **b** Quantification of the histological results obtained using the modified OARSI score; there was a significant statistical difference between surgical sides and control sides in both models. No difference was observed between the two models. Values are the mean ± SD, * = *P* < 0.05 versus controls

Representative sections of the control, fracture/dislocation and fracture-alone rats are shown in Fig. 4a. Cartilage degeneration was detected in the ankle joint, including the distal tibia and talus cartilage. In both models, OA lesion was more severe in the center cartilage than that in the peripheral parts. The summed ankle joint scores were 12.45 ± 4.01 in the dislocation model (mean ± SD, *n* = 11), and 1.73 ± 1.10 in its contralateral controls (*t* = 9.512, *P* < 0.05); while it was 11.45 ± 2.81 in the fracture model and 1.27 ± 0.90 in its contralateral controls (*t* = 9.028, *P* < 0.05). However, there was no significant statistical difference detected in the summed ankle joint scores of dislocation and fracture-alone models (*F* = 0.970, *P >* 0.05, Fig. 4b).

### Immunohistochemistry

**Fig. 5:**
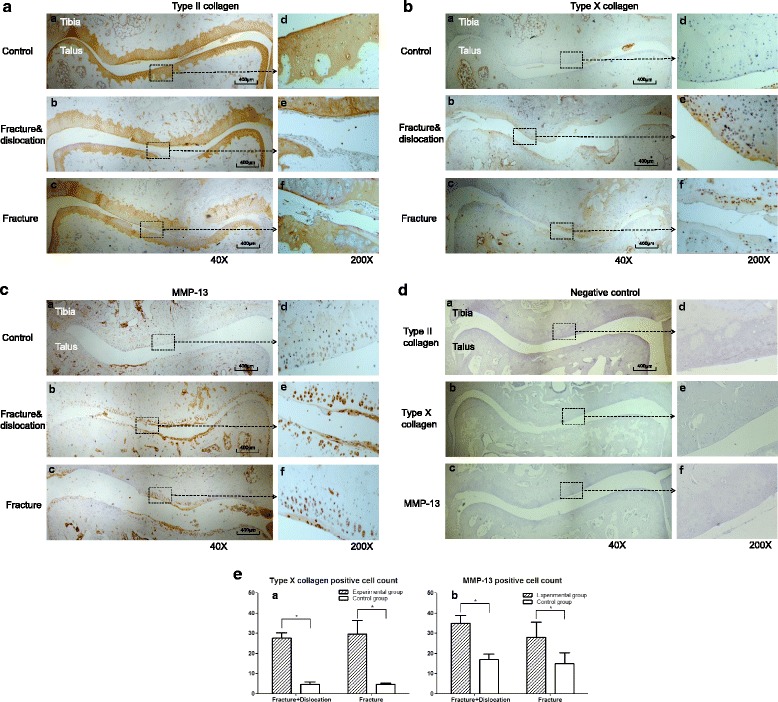
OA biomarkers were detected by immunohistochemistry. **a** Type II collagen staining was lower in the fracture/dislocation group and in the fracture alone group than in the control group. **b** Strong type X collagen staining was detected in both experimental groups. **c** Strong MMP-13 staining was detected in both experimental groups. **d** Negative control. **e** Cell counting results indicated that the number of type X collagen and MMP-13 positive cells was increased in experimental groups than control groups. Values are the mean ± SD, * = *P* < 0.05 versus controls

There was diminished type II collagen staining in the fracture/dislocation and the fracture alone groups than that in their contralateral control ankles. Strong Type X collagen and MMP-13 staining was detected both in the fracture/dislocation and in the fracture alone groups compared with their contralateral control ankle joints (Fig. 5a-5d). There were 27.67 ± 2.52 type X collagen positive cells in the fracture/dislocation group (mean ± SD, *n* = 3) and 5.00 ± 1.00 in its contralateral control group (*t* = 8.636, *P* < 0.05); as well as 29.67 ± 6.66 in fracture alone group and 4.67 ± 0.58 in its contralateral control group (*t* = 9.525, *P* < 0.05). There were 35.00 ± 4.00 MMP-13 positive cells in the fracture/dislocation group and 17.00 ± 2.65 in the contralateral control group (*t* = 7.152, *P* < 0.05); while there were 28.00 ± 7.55 in the fracture alone group and 15.00 ± 5.29 in the contralateral control group (*t* = 5.166, *P* < 0.05). However, there were no significant statistical differences detected between the fracture/dislocation and the fracture alone group (*F* = 0.1330, 1.365 respectively, *P* > 0.05, Fig. 5e).

### Real-time quantitative PCR (qPCR)

**Fig. 6:**
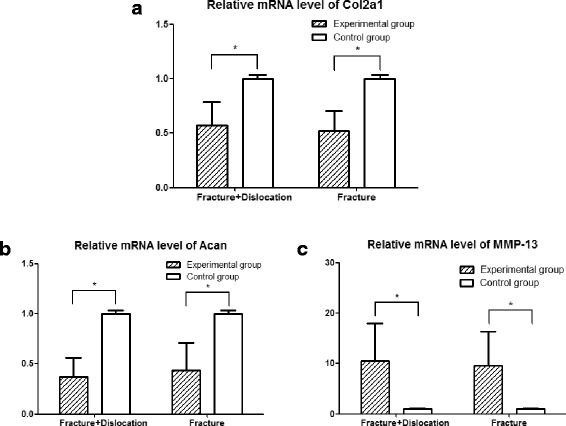
Enhanced catabolism gene expression from ankle OA cartilage detected by qPCR. The qPCR results revealed that the both experimental groups had low levels of mRNA of Col2a1 (**a**) and Acan (**b**) and increased the level of mRNA of MMP-13 (**c**) compared with the contralateral control. Values are the mean ± SD, * = *P* < 0.05 versus controls

The qPCR results indicated that both OA models had lower levels of mRNA for Col2a1 (*n* = 6) and Acan (*n* = 6), and higher levels of mRNA for MMP-13 (*n* = 6) compared with their contralateral controls (Fig. 6).

## Discussion

Approximately 1% of the world’s adult population is affected by joint pain and disability resulting from ankle OA [8]. Although knee OA has been surveyed thoroughly, the diagnosis and treatment of ankle OA may be different from those of knee OA due to the difference of metabolism, articular surface thickness and biomechanical properties [16, 22]. In healthy cadaver joints, ankle chondrocytes had increased proteoglycan (PG) and collagen synthetic rates when compared with knee chondrocytes [23]. When fibrillations and fissuring occur on the cartilage surface during early OA, markers of collagen synthesis and aggrecan turnover are increased in the ankle, but down-regulated in the knee; while markers of collagen degradation are higher in the knee than that in the ankle [24]. Ankle chondrocytes are more resistant to the effects of interleukin-1 or fibronectin fragments than those of knee cartilage, and are able to reverse their effects under the influence of BMP-7 [25]. Furthermore, ankle cartilage is significantly thinner (1–1.45 mm) than knee cartilage (3–6 mm). Joints with higher congruency appear to have thinner cartilage and have a lower incidence of osteoarthritis than noncongruent joints such as the knee [26]. In addition, ankle cartilage has a higher dynamic stiffness and compressive modulus than knee cartilage in compression, that is, ankle cartilage is more resistant to compressive loads [26].

Several OA models are available for knee OA study such as anterior cruciate ligament transection (ACLT) and destabilization of the medial meniscus (DMM), but only two ankle PTOA models have been established recently in mouse (by medial and lateral ligament resection) and mini-pig (by fracture) [27–29]. The ankle joint of mouse ligament resection model is too small to collect SF and cartilage for biomarker studies using ELISA and gene arrays using qPCR. The mini-pig model is expensive for most research groups and is not suitable for drug screen. However, the rat models have the advantages of being low cost and relative large size of joints to collect SF lavage and cartilage, as well as genetically similar within a specific breed strain, and amenable to genetic manipulation [16]. Furthermore, the anatomical and histological features of human and rodent ankle joints are comparable [28]. Therefore, it is necessary to create an innovative ankle fracture OA model for ankle OA research in the field.

Valderrabano and his colleagues showed that ankle PTOA was seen in 78% of 406 ankle OA cases; among these, malleolar ankle fractures accounted for 39% [8]. Moreover, ankle PTOA induced by fracture with dislocation is also common in clinic. Therefore, it is necessary to compare whether there is a difference between the medial malleolus fracture with dislocation injury and the medial malleolus fracture alone. Our results indicated that both the malleolar fracture models with and without dislocation/reduction were healed at 8 weeks post fracture. Based on our pilot study, we found that the 37 degree of the angle fixator created a stable fracture model located at medial 1/3 tibiotalar joint. Our results indicated that these fracture models were stable and no fixation was required. All animal fractures were completely healed without ankle joint deformity at 8 weeks after the fracture. Histology data determined by Safranin-O staining demonstrated that OA changes were similar to changes in human ankle and knee OA, including cartilage fibrillation, rough articular surface, decrease of Acan, matrix and chondrocyte numbers [17, 24, 28, 30, 31]. Increased MMP-13 and type X collagen as well as decreased type II and Acan were further detected by FMT, immunohistochemistry, qPCR and ELISA respectively. Noticeably, the fracture of medial malleolus resulted in mild to moderate OA cartilage lesions. The lesions were primarily located on the central weight-bearing region of the tibiotalar joint with a rare subchondral sclerosis. Compared with our model, the loss of cartilage and subchondral sclerosis are severe and common in the mouse ligament transection ankle OA model [28]. Our model resembles the slowly-progressive human ankle OA and should allow for evaluation of target drugs studies.

There are a few potential limitations to our study. Firstly, we used the contralateral ankle joints as controls instead of sham injured joints and unoperated joints control. The surgically-induced gait alteration may occur in the contralateral side as a result of altered loading. However, our surgical results are significantly more evident than the contralateral limbs. Nevertheless, future studies should add additional sham control groups as an ideal control. Secondly, the growth plates close at skeletal maturity and longitudinal growth ceases in adult human while the rats maintain a growth plate into old age [32]. Although this provides the potential for continued longitudinal growth, in reality bone growth ceases after a certain time (the rate of growth increases before first 5 weeks, then declines at 11.5–13 weeks, ceases until 26 weeks) [32]. Despite of all this, the difference between rodents and human should not affect our results.

The results of this study suggest that the two models can successfully induce OA, but the differences between them are not significant. Compared with the mouse ankle OA models created by transecting several ankle ligaments, our rat fracture OA model is more relevant to human ankle OA and allows for the collection of enough cartilage tissue and SF lavage for gene and inflammation biomarker analyses. Furthermore, compared with the mini-pig model, our model will be beneficial for rapid screening of targets drug with low cost.

## Conclusions

We have successfully established two rat ankle PTOA models induced by malleolus fracture. Although the OA changes observed in the two ankle OA models are similar to the changes that occur in human ankle OA cartilage, we recommend the fracture alone model as it is simpler and there is no significant difference between the two models. Thus, our ankle PTOA models will accelerate ankle OA research in the future, especially for ankle OA induced by the fracture injury.

## Abbreviations

Acan: aggrecan
ACLT: anterior cruciate ligament transection
BMP: bone morphogenetic protein
DMM: destabilization of the medial meniscus
ELISA: enzyme-linked immunosorbent assay
FAC: fibronectin-aggrecan complex
FMT: fluorescence molecular tomography
MMP-13: matrix metalloproteinase-13
OA: osteoarthritis
OARSI: Osteoarthritis Research Society International
PBS: phosphate buffer saline
PTOA: post-traumatic OA
qPCR: real-time quantitative PCR
ROI: region of interest analysis
SF: synovial fluid
